# Computational Modeling of Electrophysiology and Pharmacotherapy of Atrial Fibrillation: Recent Advances and Future Challenges

**DOI:** 10.3389/fphys.2018.01221

**Published:** 2018-09-04

**Authors:** Márcia Vagos, Ilsbeth G. M. van Herck, Joakim Sundnes, Hermenegild J. Arevalo, Andrew G. Edwards, Jussi T. Koivumäki

**Affiliations:** ^1^Computational Physiology Department, Simula Research Laboratory, Lysaker, Norway; ^2^Department of Informatics, University of Oslo, Oslo, Norway; ^3^Center for Cardiological Innovation, Oslo, Norway; ^4^BioMediTech Institute and Faculty of Biomedical Sciences and Engineering, Tampere University of Technology, Tampere, Finland; ^5^A.I. Virtanen Institute for Molecular Sciences, University of Eastern Finland, Kuopio, Finland

**Keywords:** atrial fibrillation, computational modeling, drug therapies, *in silico* drug screening, pathophysiology, pharmacology, pharmacodynamics

## Abstract

The pathophysiology of atrial fibrillation (AF) is broad, with components related to the unique and diverse cellular electrophysiology of atrial myocytes, structural complexity, and heterogeneity of atrial tissue, and pronounced disease-associated remodeling of both cells and tissue. A major challenge for rational design of AF therapy, particularly pharmacotherapy, is integrating these multiscale characteristics to identify approaches that are both efficacious and independent of ventricular contraindications. Computational modeling has long been touted as a basis for achieving such integration in a rapid, economical, and scalable manner. However, computational pipelines for AF-specific drug screening are in their infancy, and while the field is progressing quite rapidly, major challenges remain before computational approaches can fill the role of workhorse in rational design of AF pharmacotherapies. In this review, we briefly detail the unique aspects of AF pathophysiology that determine requirements for compounds targeting AF rhythm control, with emphasis on delimiting mechanisms that promote AF triggers from those providing substrate or supporting reentry. We then describe modeling approaches that have been used to assess the outcomes of drugs acting on established AF targets, as well as on novel promising targets including the ultra-rapidly activating delayed rectifier potassium current, the acetylcholine-activated potassium current and the small conductance calcium-activated potassium channel. Finally, we describe how heterogeneity and variability are being incorporated into AF-specific models, and how these approaches are yielding novel insights into the basic physiology of disease, as well as aiding identification of the important molecular players in the complex AF etiology.

## Introduction

Atrial fibrillation (AF) is a complex and multifactorial disease and the most common sustained cardiac arrhythmia, afflicting about 2% of the population. Age is the most powerful predictor of risk: approximately 5% of 65-year-olds and 10% of 75-year-olds suffer from AF (Heeringa et al., 2006). AF is already a pervasive disease carrying an immense socioeconomic burden, and with increasing life expectancy both the human and economic costs are growing rapidly: AF prevalence in the European population is projected to increase to 3% by 2030 (Zoni-Berisso et al., 2014). Although rhythm control strategies are available, these are inadequate and there is at present an unmet need for safe and effective antiarrhythmic therapy for AF (Ehrlich and Nattel, 2009). Since 2010, the European Medicines Agency has not authorized any new drugs for treatment of AF. The most prominent explanations for this lack of new medicine are the limited understanding of this multi-etiological and progressive disease, as well as the challenge of designing compounds that are strongly specific for atrial rather than ventricular targets. As a result, the development of novel pharmacological therapies is necessarily coupled to a thorough understanding of the basic etiology and physiological mechanisms of AF.

Unlike most episodes of ventricular arrhythmia, which must either be terminated or are lethal, AF does not have immediate catastrophic consequences, and short episodes of self-terminating AF are often asymptomatic and go undetected. This allows prolonged AF episodes to drive pro-arrhythmic remodeling across all levels of physiology (Schotten et al., 2011), as is succinctly captured by the phrase “AF begets AF” (Wijffels et al., 1995). In turn, this remodeling allows the mechanisms and complexity of AF to be richer than ventricular arrhythmia and causes treatment to be a moving target as the disease progresses from paroxysmal (pAF) to chronic (cAF) stages.

Both ectopic activity and the generation of a vulnerable substrate are accepted contributors to AF initiation and maintenance, although their respective contributions are thought to change as disease progresses. Triggering events are generally thought to play a more prominent role in pAF than at later stages when gross tissue-level remodeling is widespread. A range of evidence has led to this general perspective, but some key observations include: (1) prominent focal initiation of spontaneous episodes of pAF near the pulmonary vein (PV) junctions in patients (Haïssaguerre et al., 1998), (2) the absence of major alterations to action potential (AP) morphology and the excitable tissue gap in pAF (Diker et al., 1998; Voigt et al., 2013b), (3) elevated frequency of cellular triggering events (Voigt et al., 2012, 2013b).

**FIGURE 1:**
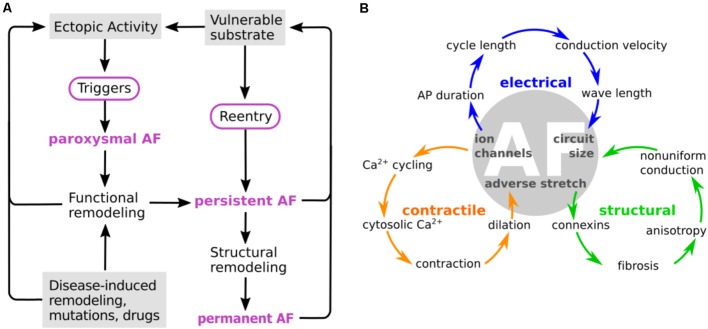
Characteristic processes and causalities at the different stages of atrial fibrillation (AF). **(A)** AF is usually triggered by ectopic activity that act as “drivers” of electrical activation and override the normal sinus node pacemaking activity. The fast pacing induced by the ectopic activity initiates electrical and structural remodeling in the atria, which enhances cellular excitability of the atria, reduces conduction velocity, increases tissue heterogeneities, and creates fibrotic regions that act as reentry anchor points. All these changes contribute to creating a vulnerable substrate that render the atria more prone to arrhythmias, as AF progresses from paroxysmal to permanent, in a process commonly termed as “AF begets AF.” **(B)** Remodeling of calcium and potassium channels leads to action potential (AP) shortening, which shortens cycle length and wavelength. Ion channel remodeling can also affect conduction velocity. Remodeling of calcium channels and intracellular calcium cycling proteins attenuates changes in cytosolic calcium, which leads to reduced contractile function and promotes atrial dilatation. This results in adverse stretch, which further causes fibrosis and connexin remodeling. Structural changes lead to non-uniform conduction, which together with electrical remodeling reduces the circuit size of electrical activation. From Allessie et al. (2002). Copyright 2012 by Oxford University Press. Adapted with permission.

As AF progresses, electrical and structural remodeling becomes pronounced, and characteristic changes to conduction and refractoriness leave the atrial myocardium more vulnerable to reentrant circuit formation (Nattel and Harada, 2014). AP duration (APD) and the effective refractory period (ERP) are consistently shortened in cAF (Iwasaki et al., 2011; Skibsbye et al., 2016), conduction is slowed (Lalani et al., 2012; Zheng et al., 2016), and the threshold for alternans induction, a key component of vulnerable substrate generation, is reduced (Narayan et al., 2011). Electrical remodeling exacerbates regional heterogeneities and promotes dispersion of refractoriness. Additionally, formation of fibrotic regions, collagen patches, and fibroblast differentiation, as part of structural remodeling, enhances tissue anisotropy and is non-uniform throughout the atria, thus further promoting the development of a reentrant substrate. Moreover, contractile remodeling (atrial dilatation and increased wall compliance) is both a consequence and effector of AF (Schotten et al., 2003). All these identified mechanisms of progressive remodeling, resulting from recurrent rapid pacing or paroxysms of AF, generate positive feedback loops that ultimately set the conditions for sustained AF. These processes are likely to be important in determining the dynamic characteristics of reentrant circuit formation, and in certain cases may be important for understanding drug action. For example, the efficacy of class Ic antiarrhythmics depends on the dynamics inherent to spiral wave propagation (Comtois et al., 2005; Kneller et al., 2005). However, currently, we do not have sufficient understanding of the tissue-level dynamics driving AF at various stages, to focus pharmacologic design efforts on correcting specific tissue-level dynamical characteristics. For this reason, our discussion below focuses on remodeling occurring at subcellular and cellular levels and their implications in AF progression, and acknowledge that the sustaining effect of tissue-level electrical and structural remodeling causes antiarrhythmic targeting in cAF to be extremely challenging. The major components, interactions, and contributions of the characteristic processes at the various stages of disease progression are summarized in **Figure 1**.

In the following four sections, we first briefly introduce the basic aspects of AF mechanisms and their related experimental findings (see the section “Arrhythmogenic Mechanisms of AF”). We then review current computational approaches for modeling atrial physiology and AF pathophysiology (see the section “*In silico* Atrial Modeling”). We present an overview of how drug–target interactions and their outcomes have been simulated in the heart, followed by current efforts to explore novel strategies for AF drug targeting (see the section “Computational Pharmacology in AF”). Finally, in the section “Modeling Variability and Uncertainty at the Cell Level,” we describe how variability and stochasticity can be incorporated into computational models to increase their robustness and predictive power in AF drug therapy.

## Arrhythmogenic Mechanisms of AF

### Remodeling of Cellular Electrophysiology, Ultrastructure, and Calcium Handling

#### Pathological Changes to Sarcolemmal Current Carriers

**FIGURE 2:**
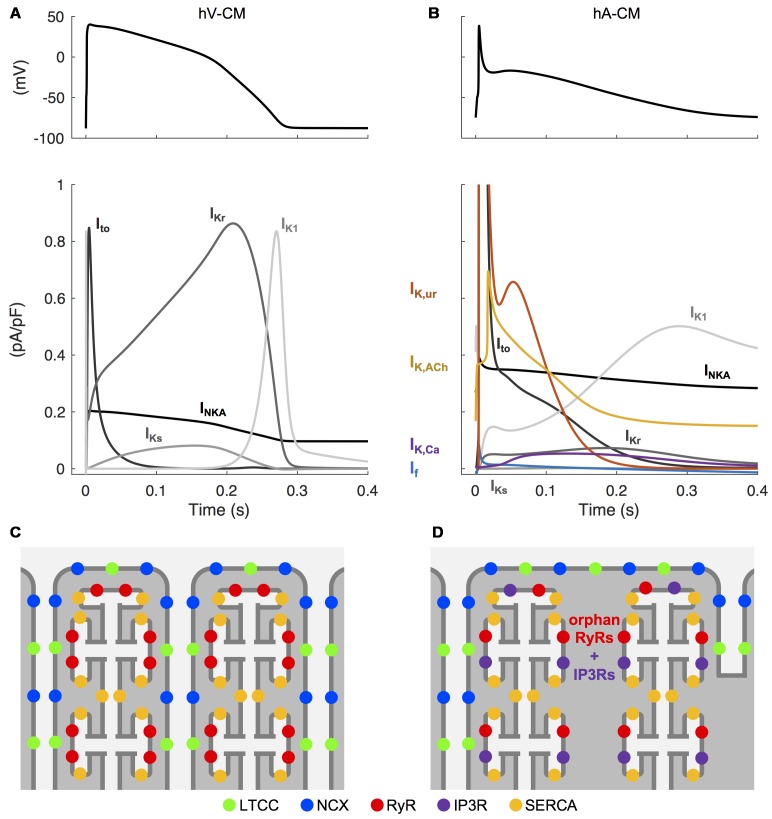
Unique electrophysiological and structural characteristics of atrial cardiomyocytes. Simulated AP in human ventricular **(A)** and atrial **(B)** CMs, as well the differences in underlying repolarizing potassium currents, obtained with the hV-CM (O’Hara et al., 2011) and hA-CM (Skibsbye et al., 2016) models, at 1 Hz pacing. Please, note that in order to visualize the weaker currents, the peaks of atrial transient outward potassium current (I_to_) (∼11 pA/pF) and the ultra rapidly activating delayed rectifier potassium current (I_Kur_) (∼4.6 pA/pF) are cut off. The atria-specific ion currents funny current (I_f_), I_Kur_, the acetylcholine-activated potassium current (I_K,ACh_), and the small conductance calcium-activated potassium current (I_K,Ca_) are highlighted with colored lines. Illustration of the relative positioning of some of the key components involved in intracellular calcium dynamics in hV-CMs **(C)** and hA-CMs **(D)**.

Human atrial cardiomyocytes (hA-CMs) exhibit a range of AP morphologies that differ markedly from those apparent in the ventricle. This is primarily due to differing expression levels of ion channel subunits, and consequent ion current densities. The atrial AP exhibits a less pronounced plateau phase, largely due to the prominent expression of the fast-activating potassium currents, particularly the ultra rapidly activating delayed rectifier current (I_Kur_), which is virtually absent in the ventricle. Atrial APs also exhibit relatively slow late repolarization (phase 3), elevated resting potential, and slower AP upstroke, all of which are strongly influenced by a reduced density of the inward-rectified potassium current (I_K1_) relative to human ventricular CMs (hV-CMs). Like I_Kur_, the small conductance calcium-activated potassium current (I_K,Ca_) is only present in hA-CMs, and it is thought to assist hA-CM repolarization, although its relative contribution remains contentious. The major differences in atrial and ventricular AP morphology and underlying ion currents are summarized in **Figures 2A,B**.

**FIGURE 3:**
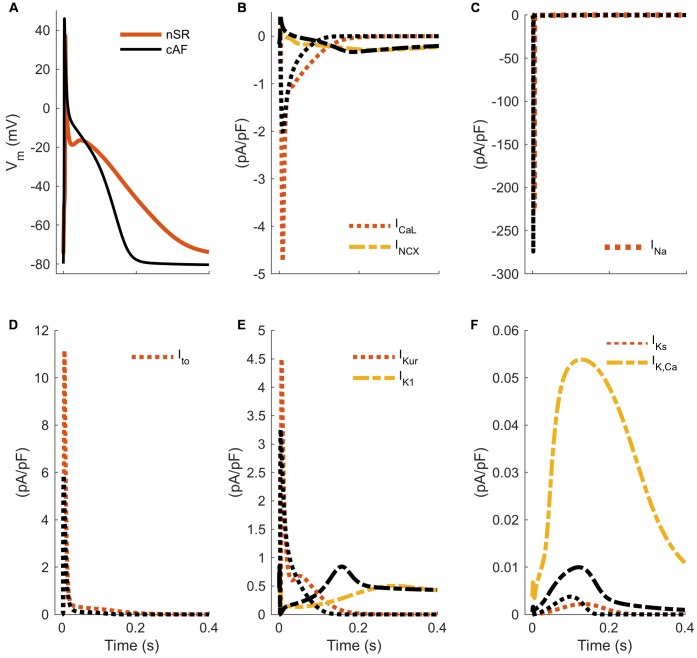
Hallmarks of altered electrophysiology of human atrial myocytes in AF. APs **(A)** and underlying ion currents **(B–F)** in normal sinus rhythm and chronic AF variants of a hA-CM model (Skibsbye et al., 2016). The cAF model variant accounts for increased/decreased ion current conductance I_Na_ (–18%), I_CaL_ (–59%), I_to_ (–62%), I_Kur_ (–38%), I_K1_ (+62%), I_Ks_ (+170%), I_K,Ca_ (–50%) and I_NCX_ (+50%), as summarized in Koivumäki et al. (2014b) and Skibsbye et al. (2016).

The pathophysiology of cAF is characterized by several prototypical changes in current expression that result in both marked deceleration of early repolarization, and acceleration of late repolarization (Schotten et al., 2011). The two most prominent molecular changes that drive these outcomes are: (1) augmentation of inward-rectified potassium currents (increase of I_K1_ expression and constitutive activity of the acetylcholine-activated inward rectifier current; I_K,ACh_), and (2) simultaneous decrease in the L-type calcium current (I_CaL_). The major counteractive changes are carried by increased sodium–calcium exchanger (NCX) expression, and reductions in the major rapidly activating outward currents, namely I_Kur_ and the fast component of the transient outward potassium current (herein simply referred to as I_to_). Together, these five alterations (I_K1_, I_CaL_, NCX, I_Kur_, and I_to_) constitute the majority of the known modulators of repolarization trajectory in cAF (**Figure 3**). Overall these effects result in the shortening of ERP, and a slightly more negative resting membrane potential (Ravens et al., 2014; Skibsbye et al., 2014, 2016), both of which expand the window for reentrant excitation. However, as discussed further below, they are accompanied by a range of changes to cellular ultrastructure and to the function of major calcium- and sodium-handling proteins, such that predicting the integrated outcomes from any subset of changes is non-trivial and necessitates quantitative approaches.

#### Ultrastructural Contributions to AF Pathogenesis

Although there is important species-specificity of atrial CM ultrastructure, it has been generally observed that healthy atrial CMs exhibit a varied and relatively sparse membrane ultrastructure compared to ventricular CMs. This results in important baseline differences in excitation–contraction coupling. Most prominently, atrial CMs have a less developed T-tubule network (Dibb et al., 2013), particularly in rodents, as illustrated in **Figures 2C,D**. This morphological difference has implications for intracellular calcium diffusion. In the absence of T-tubules, Ca^2+^ enters the cells largely from the periphery, and thus must diffuse centripetally to engage the contractile machinery. Correspondingly, the localization of Ca^2+^ handling proteins is very different in ventricular and atrial CMs. In hA-CMs, as in hV-CMs, L-type Ca^2+^ channels interact with clusters of sarcoplasmic reticulum (SR) Ca^2+^ release channels (RyRs; ryanodine receptors) located in the junctional SR to trigger Ca^2+^-induced Ca^2+^ release (CICR). However, in hA-CMs, a higher proportion of RyR clusters are concentrated in non-junctional SR, and this is a distinguishing structural characteristic. These orphan or non-junctional RyRs contribute to the fire-diffuse-fire propagation of Ca^2+^, which is augmented by inositol 1,4,5-trisphosphate receptors (IP3Rs) that are also embedded in the SR membrane (Lipp et al., 2000; Yamada et al., 2001; Zima and Blatter, 2004; Li et al., 2005; Wullschleger et al., 2017). The importance of IP3Rs is generally greater in atrial than ventricular CMs – in ventricle, they are generally only observed in disease states, such as heart failure (Go et al., 1995). These features of the calcium signaling system fundamentally alter the essential structure-function relationships governing calcium handling in atrial versus ventricular CMs, where the extensive and highly organized T-tubule network shortens the diffusion distances so that fast and uniform CICR is possible. The physiological outcome for the atrial CM is a slower rise phase of the intracellular Ca^2+^ transient (CaT) (Hatem et al., 1997; Greiser et al., 2014) and contractile force (Frisk et al., 2014), and ∼100 ms delayed CaT at the center of the CM comparatively to the periphery (Hatem et al., 1997; Tanaami et al., 2005; Greiser et al., 2014), resulting from spatial (particularly centripetal) propagation of intracellular Ca^2+^ during atrial systole.

This unique membrane ultrastructure of atrial CMs is now also thought to contribute to AF pathogenesis. Recently, it has been shown that T-tubule density in atrial cells is reduced in sheep and canine models of AF (Lenaerts et al., 2009; Wakili et al., 2010); however, supporting human atrial data is lacking. The putative loss of T-tubules may lead to contractile dysfunction, but is also strongly implicated in arrhythmogenesis. In particular, the increased spatial heterogeneity in subcellular Ca^2+^ signaling has been shown to promote CaT and APD alternans (Gaeta et al., 2009; Li et al., 2012), and incomplete excitation–contraction (E–C) coupling (Greiser et al., 2014). Reorganization of RyR clusters adds a further dimension to AF-related ultrastructural remodeling. It has been shown to be associated with more frequent Ca^2+^ sparks in a sheep model of cAF, and is thought to increase the probability of the propagating Ca^2+^ release underlying arrhythmogenic calcium waves (Macquaide et al., 2015). However, there are no human data available to corroborate the possible change in organization of RyRs in AF patients. Thus, additional structural and functional data from patients would be valuable for understanding the functional role of structural degradation in this disease.

Cell dilation/hypertrophy is also a common finding in cAF patient samples, where increases of 12% (Neef et al., 2010) and 16–54% (Schotten et al., 2001; Neef et al., 2010; Corradi et al., 2012) have been reported for length and diameter, respectively. In line with these findings, cell surface area in patients with cAF was reported be ∼40% larger (Wouters et al., 2000). The increased cell volume and diameter reduce CaT amplitude and slow centripetal Ca^2+^ diffusion, respectively (Koivumäki et al., 2014b). As hA-CMs are likely to have very few (if any) T-tubules in cAF, slower Ca^2+^ diffusion is thought to exacerbate dyssynchrony of the AP and CaT, thus potentially contributing to alternans. At the tissue level, increased capacitance of CM membrane causes conduction slowing (Oliveira et al., 2015).

As mentioned above, tissue-level remodeling, inflammatory signaling, and mechanical dysregulation also make a major contribution to AF pathology, particularly in the advanced stages of disease. We mention these aspects briefly here, but the remainder of this article will focus on classical electrophysiologic and ionic mechanisms of AF, particularly those targeted for acute cardioversion early in disease development. Reduced I_CaL_ in cAF promotes contractile dysfunction and atrial dilatation (atrial stretch). These mechanical perturbations are thought to be a major contributor to the widespread deposition of interstitial collagen, lateralization of gap junctions (connexin remodeling), and proliferation of myofibroblasts and potentially adipocyte infiltration observed in many animal models of chronic disease (Ravelli and Allessie, 1997; Schotten et al., 2003; Lau et al., 2017). While these characteristics are widely thought to be similarly prominent in humans, corroborating data remain relatively sparse because *in vivo* measures are technically challenging. Functional indicators (e.g., complex fractionated atrial electrograms) have often been used as primary measures of fibrosis, although gadolinium-enhanced MRI protocols have also been shown capable of quantifying *in vivo* differences between paroxysmal and more advanced disease (Daccarett et al., 2011). These changes in atrial tissue structure have profound consequences for tissue conductivity, wave propagation, and potential for reentry, and are thus likely to pose an insurmountable challenge to pharmacotherapy in later disease stages. For this reason, interventions targeting the suppression of the signaling pathways that results in these gross changes to atrial structure, have recently become an area of substantial interest (Nattel and Harada, 2014).

#### Role of Remodeled Calcium Homeostasis in AF

Alterations to calcium handling are intrinsically linked to the ultrastructural changes described above, but further remodeling of expression or regulation of the major intracellular transporters is also likely to contribute. In general, the role for these mechanisms in AF, particularly pAF, has become well supported in recent years, and ion transporters involved in calcium handling and their regulatory proteins seem to be promising targets for drug therapy of AF. As mentioned above, cAF is associated with increased NCX expression in patients (Gaborit et al., 2005; El-Armouche et al., 2006; Neef et al., 2010). There is also strong evidence of an increased coupling gain between intracellular Ca^2+^ load and I_NCX_ in cAF (Grandi et al., 2011; Voigt et al., 2012) and larger I_NCX_ amplitudes have also been reported in cAF patient samples (Christ et al., 2016). The data on altered RyR function in AF is less conclusive. Increased RyR activity has been reported in cAF patients (Neef et al., 2010; Voigt et al., 2012), whereas, RyR expression has been reported to be both reduced (Ohkusa et al., 1999; Oh et al., 2010) and unchanged (Shanmugam et al., 2011; Voigt et al., 2012) in cAF patients. Hyperphosphorylation of RyRs has been reported to increase their Ca^2+^ sensitivity and open probability, increasing Ca^2+^ leak from the SR into the cytosol (Vest et al., 2005; Neef et al., 2010; Voigt et al., 2012). One further player in the game of calcium remodeling is the SR Ca^2+^-ATPase (SERCA), which pumps Ca^2+^ back into the SR from the cytosol. SERCA function is regulated by two inhibitory proteins: phospholamban and sarcolipin, and the phosphorylation levels of these regulatory proteins has an impact on the amplitude of the CaT and SR Ca^2+^ load. Reduced SERCA protein expression accompanied by increased activity was found in both pAF patients (Voigt et al., 2013b), while a rabbit model of rapid atrial pacing has shown remodeling-induced reduction in expression levels of SERCA with unchanged activity (Greiser et al., 2014). Although SERCA plays an important role in the modulation of SR Ca^2+^ load and, indirectly, in the extent of arrhythmogenic Ca^2+^ leak, there is currently no published *in vitro* human data on the AF-related change in function of SERCA, and the protein expression data is not conclusive.

### Cellular Electrophysiologic Instability in AF

**FIGURE 4:**
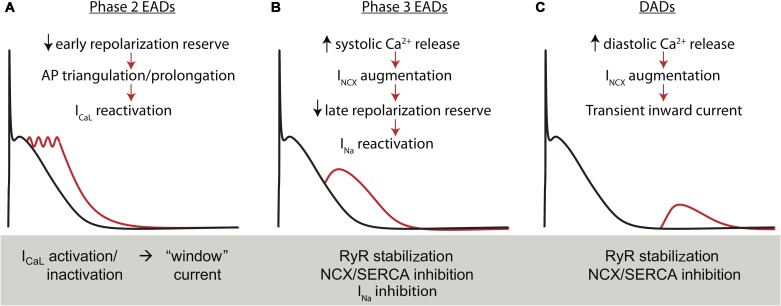
Electrophysiologic instabilities in atrial myocytes. Early afterdepolarizations in hA-CMs exhibit the same essential mechanisms as occur in ventricular cells. **(A)** Phase-2 EADs result from the kinetic interaction of I_CaL_ and the outward potassium current involving multiple K^+^ channel species. In general, these events are uncovered during reduced repolarization reserve through I_CaL_ potentiation or K^+^ channel antagonists, particularly I_Kr_ inhibitors. **(B)** A second class of EADs occurs when repolarization is disrupted during phase 3 by forward mode Na^+^-Ca^2+^ exchange. This additional I_NCX_ is a secondary effect of dis-coordinated or simply exaggerated I_CaL_-triggered SR Ca^2+^ release. Importantly these EADs generally recruit either I_CaL_ or I_Na_ as the major carrier of the inward current after I_NCX_ has sufficiently slowed late repolarization. **(C)** Finally, I_NCX_ is also responsible for initiating DADs secondary to spontaneous SR Ca^2+^ release during atrial diastole. Time scale for all panels is 0–1 s and voltage range from –80 to +40 mVs.

As described above, one of the proposed mechanisms of AF initiation is the generation of triggered activity in the atria in early stages of AF. These triggering events are classified as they are in the ventricle. That is, instabilities in AP repolarization are named early afterdepolarizations (EADs), and diastolic instabilities initiating from resting potential are delayed afterdepolarizations (DADs). Several of the established mechanisms of EADs and DADs are described in **Figure 4**. Because repolarization is hastened and I_CaL_ is reduced in cAF, AP triangulation is also reduced and the conditions for EAD generation via conventional I_CaL_ reactivation are generally impaired (Ming et al., 1994; Burashnikov and Antzelevitch, 2006). However, a body of literature supports that EADs initiating late in phase 3 of the AP may be important in some atrial regions and contexts, particularly focal arrhythmia initiating in the PV sleeves (Burashnikov and Antzelevitch, 2003; Patterson et al., 2006; Morotti et al., 2014, 2016). These EADs are driven by enhanced Ca^2+^ signaling, which in turn exaggerates I_NCX_, slows late repolarization, and thereby promotes I_Na_ reactivation (Morotti et al., 2014, 2016).

The decreased Ca^2+^ influx via I_CaL_, enhanced calcium extrusion due to increased NCX expression, and a leaky population of RyR, has generally been observed to result in marked depletion of the intracellular Ca^2+^ in cAF. In and of itself, this would be expected to reduce the incidence of spontaneous Ca^2+^ waves and DADs, and the ability of these diastolic events to drive focal arrhythmia. Indeed, the majority of studies support silencing of Ca^2+^ signaling as a cardioprotective mechanism and a reduced role for spontaneous Ca^2+^ release in cAF (Schotten et al., 2007; Christ et al., 2014; Greiser et al., 2014; Koivumäki et al., 2014b). However, opposite findings have also been reported in hA-CMs from cAF patients (Voigt et al., 2012). Importantly, rather than being reduced, SR Ca^2+^ load was maintained in that study, and thus the elevated RyR activity and NCX expression readily translated to increased Ca^2+^ waves and DADs. Data from patients in pAF suggest that SR Ca^2+^ load is either not depleted (Hove-Madsen et al., 2004), or may in fact be exacerbated at these early stages of disease (Voigt et al., 2013b). Thus, the conditions explaining the observed increases in magnitude and frequency of spontaneous Ca^2+^ waves are more obvious and consistent. Viewing this collection of studies together, the most parsimonious interpretation is that the molecular drivers of increased Ca^2+^ wave frequency (RyR hyperphosphorylation, possibly increased SERCA activity) may precede those that strongly deplete intracellular Ca^2+^ (NCX expression). Thus, the increase in spontaneous Ca^2+^ release observed early in AF may be lost as the delayed molecular adaptations, particularly increased NCX expression, act to shift Ca^2+^ flux balance toward extrusion, thus depleting the Ca^2+^ store and silencing Ca^2+^ signaling, even during tachycardia (Greiser et al., 2014). This conceptual model of how Ca^2+^-driven diastolic instability develops during AF is largely hypothetical, and further characterization of the specific temporal development of these molecular and functional maladaptations during disease is highly desirable.

Another proposed mechanism of triggered diastolic activity in the atria has stemmed from the discovery of expression of hyperpolarization-activated cation channels (HCN), carriers of the pacemaker current (I_f_), in the left atrial appendage (Zorn-Pauly et al., 2004; Scheruebel et al., 2014). Furthermore, I_f_ properties are altered in cAF (Stillitano et al., 2013), lending weight to the hypothesis of abnormal cell automaticity as an additional mechanism of diastolic triggered activity in the remodeled myocardium. HCN channels could, therefore, constitute a novel potential target for antiarrhythmic drug therapy. However, the lack of conclusive experimental human data thus far has rendered this mechanism a less attractive option for pharmacotherapy discovery.

Finally, the role of APD alternans in driving AF initiation is increasingly becoming appreciated after the observation that it immediately precedes AF in patients (Narayan et al., 2011). While the mechanisms capable of driving APD alternans are diverse, dynamic, and interactive, a growing body of evidence suggests that the proximal driver at the cellular level is a period 2 instability in Ca^2+^ cycling (Gaeta et al., 2009). In ventricular CMs this form of instability was initially thought to be driven by a kinetic mismatch in SR Ca^2+^ reuptake leading to variable refractoriness of Ca^2+^ release at high pacing frequencies (Diaz et al., 2004). However, more recently the role of subcellular heterogeneities in Ca^2+^ dynamics has emerged as a central aspect to the link between APD and Ca^2+^ transient alternans (Shiferaw and Karma, 2006; Gaeta et al., 2009, 2010; Gaeta and Christini, 2012). The intrinsic variability in atrial CM ultrastructure would be expected to promote these behaviors, particularly in AF, and this relationship between structure and dysfunction in AF requires stronger investigation by computational approaches.

## *In Silico* Atrial Modeling

### Existing hA-CM Models and AF Model Variants

**FIGURE 5:**
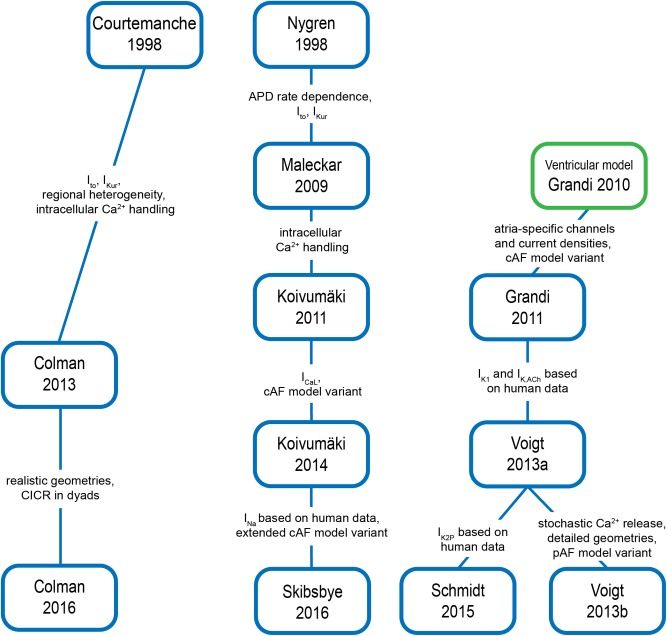
Overview of hA-CM model lineages and most important changes implemented in model iterations. References: Courtemanche et al. (1998), Nygren et al. (1998), Maleckar et al. (2009b), Grandi et al. (2010), Grandi et al. (2011), Koivumäki et al. (2011), Colman et al. (2013), Voigt et al. (2013a,b), Koivumäki et al. (2014b), Colman et al. (2016), Skibsbye et al. (2016).

Models for cardiac cellular electrophysiology and ion dynamics have been developed for more than five decades (Noble, 1962), and the first atria-specific hA-CM models were published by Courtemanche et al. (1998) and Nygren et al. (1998). These model lineages have been retroactively extended with novel features, and new models have also been introduced as shown in **Figure 5**. The Courtemanche, Nygren–Maleckar–Koivumäki and Grandi model lineages, were benchmarked in detail (Wilhelms et al., 2013), and shown to be based on varying datasets and assumptions. As Wilhelms et al. (2013) reported, there are substantial differences in AP and CaT morphology, and rate adaptation properties among these models. For example, the AP repolarization in the Courtemanche model depends more on I_Kr_ and less on I_Kur_ compared to the other models. The Nygren model has a substantially larger contribution of the I_Ks_ current. Furthermore, several models include ion currents not incorporated in the others. For example, I_K,ACh_ (Maleckar and Grandi models); I_f_ (Koivumäki model); plateau potassium current, the Ca^2+^ dependent chloride current and background chloride current (Grandi model).

With accumulating experimental (human) data supporting the unique characteristics of atrial Ca^2+^ handling and its role in AF pathophysiology and arrhythmogenesis, the foundational hA-CM models have been updated to particularly include more complex intracellular Ca^2+^ signaling and ion channel localization. To account for the centripetal diffusion of calcium due to the lack of T-tubules in hA-CMs, Voigt et al. (2013b) extended the Grandi model with a spatial representation of Ca^2+^ handling based on longitudinal and transverse division of the intracellular space, and included stochastic RyR gating. Colman et al. (2016) also presented an atrial model with spatial representation of the calcium handling system to assess the role of variable T-tubule density on intracellular calcium waves and alternans. These efforts have generally attempted to approach more realistic Ca^2+^ handling representations by drawing on data describing the T-tubule structure in particular. For instance, it may be possible to replicate the approach now being taken in ventricular CMs, where realistic SR and T-tubule geometries resolved by serial scanning electron microscopy have made it possible to reconstruct large sections of the cell directly from data (Colman et al., 2017). When applied to atrial CMs, this approach may provide a more realistic basis for simulating the effects of subcellular structure on macroscopic E–C coupling and arrhythmogenesis.

In addition to recapitulating physiology of healthy hA-CMs, all the above-mentioned cell models have variants to mimic cellular remodeling related to AF. The principle of ‘AF begets AF’ (Wijffels et al., 1995) emphasizes the need to represent the pathophysiological changes at different stages of AF progression with dedicated models. So far, the only pAF model variant has been published by Voigt et al. (2013b), accounting for early dysregulation of SR Ca^2+^ release and enhanced uptake, with no significant changes to sarcolemmal current carriers, and AP morphology. Conversely, cAF involves a much more advanced and complex remodeling (Schotten et al., 2011), which has been implemented in the *in silico* models to varying degrees of detail. The vast majority of cAF model variants have focused on electrical remodeling as distinct from remodeling of subcellular structure and Ca^2+^ handling machinery. These efforts have generally included the decreased I_to_, I_CaL_ and I_Kur_, and increased I_K1_, as described in the section “Arrhythmogenic Mechanisms of AF.” More recently, cAF models that also account for the remodeling of intracellular Ca^2+^ handling have been developed (Grandi et al., 2011; Colman et al., 2013; Voigt et al., 2013a; Koivumäki et al., 2014b). Furthermore, AF-related structural remodeling, specifically cell dilation, has been represented in one hA-CM model (Koivumäki et al., 2014b). First steps in accounting for the role of changes to regulatory signaling have also been taken by Grandi et al. (2011), who showed dramatic APD shortening as a result of parasympathetic activation of I_K,ACh_. However, the overly simplified I_to_ – I_CaL_ – I_Kur_ – I_K1_ approach of cAF modeling is still commonly used. As the accumulating experimental evidence suggests a central role for altered E–C coupling and intracellular Ca^2+^ handling in AF pathophysiology, a greater emphasis should be put on these components in future modeling studies.

*In silico* hA-CM models are comprehensive tools, complementing the *in vitro* experiments, for increasing the understanding of AF mechanisms and discovering potential pharmacological targets. The diversity of hA-CM models adds a layer of complexity to modeling of pharmacodynamics, as the outcome of pharmacological interventions *in silico* will vary between different models. This will be discussed in detail in the section “Computational Pharmacology in AF.” As the physiological accuracy and robustness of atrial CM models have improved over the years, and continues to progress, so do their utility in higher dimensional and organ scale simulations, as discussed further below.

### 1D and 2D Models of Electrical Conduction in the Atria

#### Modeling Electrical Propagation in Tissue

Early observations of electrograms during AF revealed the presence of chaotic activity in the atria. Recent technological advancements in high-resolution AF mapping have shown that AF is maintained by one or multiple spiral waves or rotors, which may be stationary or meander around anatomical structures (Guillem et al., 2016). Several studies have further supported that AF is maintained by high-frequency reentrant activity, compatible with the mother rotor hypothesis, as recently reviewed (Waks and Josephson, 2014; Guillem et al., 2016). However, several open questions still remain regarding the exact dynamical drivers of AF. Computational models of electrical propagation in the atria have contributed to elucidating the mechanisms of arrhythmia by enabling the simulation of electrical propagation in the heart through simplified models of single cell myocyte networks, mainly in the form of 1D and 2D architectures representing atrial fibers or patches of atrial tissue.

Characteristics of electrical activation in the myocardium, such as conduction velocity (CV), ERP, CV restitution, and APD restitution are known to modulate impulse propagation, with CV and refractoriness largely determining rotor dynamics and reentry stability (Sánchez et al., 2012). This commonly cited conceptual model is termed “leading circle” reentry, and states that a reentrant wavefront is permitted to follow a circular path of minimal length equal to the wavelength (CV^∗^ERP), with the core remaining continuously refractory. Reported values of CV measured in the atria lie between ∼50 to ∼120 cm/s (Dössel et al., 2012), and are reduced by ∼15% in AF (Feld et al., 1997). Conduction slowing and ERP shortening are two hallmarks of AF-induced remodeling, which result in reduced wavelength and higher susceptibility to reentry (Starmer et al., 1991; Nattel et al., 2008; Wakili et al., 2011; King et al., 2013). Conveniently, ERP and changes to ERP resulting from pharmacotherapy, can be implemented in 0D cell models, and CV can then be assessed by applying simple cable theory to couple such 0D implementations in series. The resulting 1D simulation frameworks are often used to arrive at basic indications of pharmacologic impacts on susceptibility to reentry, without actually permitting reentrant excitation.

At a higher level, 2D patches of tissue have been employed in simulations to reproduce the effects of structural and electrical remodeling on conduction barriers and exacerbated electrophysiological heterogeneities leading to unidirectional block and spiral wave breakup. As will be further discussed in the section “I_Na_,” these frameworks have been very important for establishing the role of spiral wave dynamics in explaining the efficacy and subtype specificity of I_Na_ blockade. Aslanidi et al. (2009b) used 2D models of the atria to study activation patterns in the absence and presence of electrical heterogeneity, independently of structural effects or conduction anisotropy. Results of 2D simulations show that APD gradients across the atria alone can reproduce different activation patterns in different regions of the atria (LA versus RA) (Aslanidi et al., 2009b). More recently, Gharaviri et al. (2017) studied the effect of transmural conduction using a dual sheet model of atrial tissue. They found that reducing the number of connections between the endo- and epicardial layers resulted in increased endo-epicardial dyssynchrony of electrical activity and in enhanced AF stability, in agreement with experimental findings in patients and animals (Verheule et al., 2013; Hansen et al., 2015).

Additionally, computational tissue models have helped elucidate the molecular mechanisms that give rise to spatially discordant alternans (SDAs), a mechanism that has been linked to the development of an arrhythmogenic substrate and increased reentry incidence (Pastore and Rosenbaum, 2000). Clinical data has shown that AP alternans precede episodes of AF in patients (Narayan et al., 2011), and another study in healthy controls and persistent AF patients showed that rapid pacing-induced SDA were associated with AF incidence, and could be terminated by verapamil administration (Hiromoto et al., 2005). These findings highlight the potential arrhythmogenic role of alternans, and the need to further elucidate the contribution of SDA to AF. Experimental studies in whole heart (Pruvot et al., 2004) and modeling studies in both 1D and 2D ventricular tissue have shown that SDA can be attributed to different mechanisms, in particular, Ca^2+^ instabilities (Sato et al., 2006, 2013), steep APD and CV restitution, (Qu et al., 2000) and tissue heterogeneities (Watanabe et al., 2001).

Because these 1D and 2D tissue models remain relatively computationally efficient, they can also be used to assess the ionic determinants that modulate conduction and rate adaptation in the atria during rapid pacing or other processes involving manipulation of the electrophysiologic steady state (Starmer et al., 2003; Krummen et al., 2012; Hunnik et al., 2016). In these contexts, extracting measures from simulated reentrant circuits allows quantitative comparisons of the impact of different ionic mechanisms and model conditions on the incidence and magnitude of AF, commonly quantified in terms of dominant frequency (DF), organization index, rotor meandering (RM), and duration of reentry.

#### Fibrosis in 1D and 2D Models

Tissue models of the atria have recently been expanded to include the effect of fibrosis in AF maintenance. Myofibroblasts, or simply fibroblasts (Fb), compose about 10–15% of myocardium volume, although they largely outnumber myocytes (Shiraishi et al., 1992). Fibroblasts can exhibit APs when electrically coupled to CMs through gap junction channels (Camelliti et al., 2004; Wang et al., 2006), and have long been recognized to play an important role in modulating the electrical function of the myocardium (Kohl and Noble, 1996; Kamkin et al., 1999; Kohl et al., 2005; Tanaka et al., 2007). The fibroblasts can act as either current sources or sinks during a myocyte excitation, disturbing normal electrical propagation, and their proliferation has also been linked to abnormal automaticity in the atria, whereby Fb-CM coupling can induce a depolarizing current during the diastolic phase and elicit APs (Miragoli et al., 2007).

In agreement with *in vitro* experimental data, modeling studies in 1D and 2D models have shown that proliferation of fibroblasts (or more generically, non-myocytes) in the atrial tissue, and their coupling with myocytes through gap junctions, lead to alterations of the AP shape, RMP, upstroke velocity and CV. The significance of the alterations, and their arrhythmogenic potential, depends on several factors, such as fibroblast density and distribution, the strength of Fb-CM coupling, and RMP of the fibroblast (Jacquemet and Henriquez, 2008; Maleckar et al., 2009a; Koivumäki et al., 2014a; Seemann et al., 2017), as reviewed in Yue et al. (2011). Additionally, Fb-CM coupling can lower or raise the APD alternans threshold, depending on whether APD is shortened or prolonged (Xie et al., 2009). Furthermore, in cAF, the hyperpolarization of the membrane potential has been shown to render the remodeled myocytes less sensitive to coupling with fibroblasts (Sánchez J. et al., 2017). In another study, the APD shortening effect of dofetilide and vardenafil was enhanced with increasing amount of coupled fibroblasts, showing the importance of including Fb-CM coupling in pharmacological modeling studies (Gao et al., 2017).

Although human data is still sparse, and the precise contribution of fibrosis to ectopic activity and reentry in AF remains poorly understood, it seems clear that fibrotic tissue is a key promoter of AF progression. Therefore, therapeutical approaches that prevent fibroblast proliferation, secretion and connexin expression, by targeting for example fibroblast ion channels and signaling pathways, could constitute a potential strategy for upstream regulation of AF progression (Yue et al., 2011).

### 3D Models of the Atria

Single cell models of atrial electrophysiology have significantly contributed to increase our understanding of the cellular mechanisms of arrhythmia and underpinning novel pharmacotherapeutic targets (Heijman et al., 2015). However, multiscale models of the atria are necessary to understand the complexity of atrial arrhythmias and capture the essential dynamics of this disease. This need is accentuated by challenges associated with obtaining reliable AF activation maps, especially in patients, which has pressed the need for more elaborate *in silico* whole atrial models. Three-dimensional (3D) models of atrial electroconduction have been developed to enable simulation of normal atrial function and arrhythmogenesis in the context of full structural complexity of the atrial geometry, and incorporating many of the regional electrical heterogeneities present in the intact organ.

Electrical heterogeneities in the atria are mainly characterized by regional variations of ion current and connexin expression. However, as human data are sparse, these regional differences are generally incorporated from studies conducted in other species, mostly canine. The complex structural heterogeneities in the atria are also challenging to accurately represent in computational models, but are believed to be important for the understanding of AF dynamics. Thus, in recent years a considerable amount of effort has been devoted to the incorporation of detailed anatomic, structural and electrophysiological information in the modeling pipeline.

#### Incorporating Heterogeneity Into 3D Models of the Atria

The first attempts to develop 3D models of the atria relied on simplistic geometries with limited anatomical detail, such as spherical surfaces (Blanc et al., 2001), or geometrical surfaces designed to resemble the atria (Harrild and Henriquez, 2000; Vigmond et al., 2003; Ruiz-Villa et al., 2009). Additionally, most of these first models did not consider regional differences in electrophysiology (Harrild and Henriquez, 2000; Blanc et al., 2001; Virag et al., 2002). In their first stage of development, 3D models of the atria were mostly focused on the role of atrial geometry and structural heterogeneity on the development of a proarrhythmic substrate (Harrild and Henriquez, 2000; Blanc et al., 2001; Virag et al., 2002). Although useful in discerning the basic mechanisms underlying atrial arrhythmias, these studies recognized the importance of incorporating electrical and more detailed structural heterogeneity into the models in order to faithfully reproduce complex arrhythmogenic patterns. Vigmond et al. (2003) presented the first atrial model, a canine model, containing all the major structural features of the atria, electrical propagation according to fiber orientation (constructed with a series of interconnected cables), AP heterogeneity, and electrical remodeling. The study provided new insight into the role of structural and electrical heterogeneity of atrial tissue on reentry and fibrillation maintenance, and confirmed the importance of including electrophysiological variations in atrial tissue models.

**FIGURE 6:**
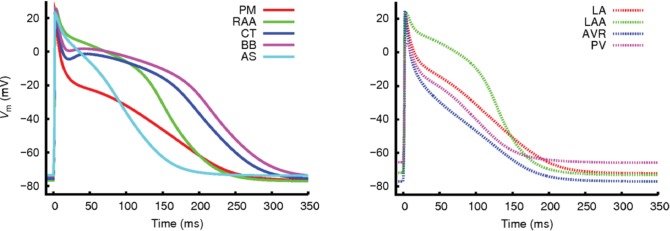
Regional differences in AP morphologies in the different atrial regions obtained with a hA-CM model. From Colman et al. (2013). Creative Commons Attribution License BY.

Since these earlier efforts, regional differences in AP morphology have typically been incorporated by varying ion channel maximum conductances and gating variables of the Courtemanche hA-CM model. **Figure 6** shows an example of different AP morphologies in the different regions of the atria modeled in this way (Colman et al., 2013). These are mainly due to differences in expression of I_Kr_, I_Ks_, I_to_, I_Kur_, I_K1_, and I_CaL_. We will not describe these regional characteristics in detail, but a comprehensive overview of current densities and APD in the different atrial regions, and of the original experimental data sources, can be found in Krueger et al. (2013). With these model variants as a baseline for electrical variation throughout the atria, it has been possible to begin understanding the role of electrophysiological heterogeneity both in normal atrial activation, and in AF arrhythmogenesis. During normal activation, the gradient in APD from the sino-atrial node (SAN) toward the atrio-ventricular node (AVN) and the left atrium (LA) (Ridler et al., 2006), is thought to facilitate conduction from the SAN toward the AVN and impede uni-directional conduction block during normal sinus rhythm. However, the role of these APD gradients in atrial arrhythmias is not fully understood, and the manner in which change associated with AF electrical remodeling contribute to arrhythmia is very complex (Colman et al., 2013). Patchy tissue heterogeneities in left versus right atria are known to promote AF initiation (Luca et al., 2015), and it has often been suggested that left-right gradients in ion current expression increase dispersion of refractoriness and thereby promote reentrant substrate (Voigt et al., 2010). However, computational studies of the effect of right-left APD gradients in a canine model has found these gradients to be a protective mechanism against reentry, while increasing the complexity of arrhythmia patterns (Ridler et al., 2006, 2011). These studies highlight the complex effect of atrial heterogeneities and the need for a systematic characterization of the role of spatial variation of cell and tissue properties in AF.

In addition to the varying AP morphology, the atria present significant regional differences in CV and fiber orientation. These differences can be represented in models by spatially varying tissue conductivities according to tensor vectors obtained from fiber direction information. Fiber direction can be obtained with rule based methods (Seemann et al., 2006; Aslanidi et al., 2011; Colman et al., 2013), based on anatomical data obtained from *ex vivo* diffusion-tensor imaging (Pashakhanloo et al., 2016), or histological slices (Butters et al., 2013; Tobón et al., 2013).

**FIGURE 7:**
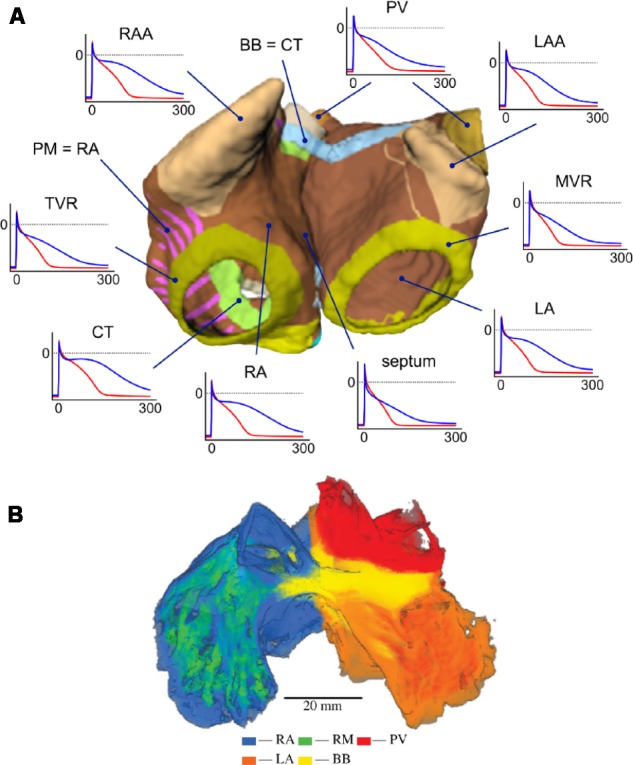
**(A)** Three-dimensional atrial model with segmented regions and corresponding APs in physiological (blue) and AF-remodeled conditions (red), obtained with the Courtemanche model. From Krueger (2013). Creative Commons Attribution License BY-NC-ND. **(B)** Regional segmentation of an atrial sheep model into right atrium (RA), left atrium (LA), pectinate muscles (PM), pulmonary veins (PV), and Bachmann’s Bundle (BB). From Butters et al. (2013). Copyright 2013 by Royal Society (United Kingdom). Reprinted with permission.

Seemann et al. (2006) published the first model implementing realistic full 3D atrial geometries with regional heterogeneity. This model incorporated heterogeneity based on both human and animal experimental data of several atrial structures: Crista terminalis (CT), pectinate muscles (PM), Bachmann’s bundle, atrial working myocardium, atrial appendage, and SAN. More recently, Krueger et al. (2013) developed an extended model with patient-specific anatomical data and additional segmentation of atrial regions: the PVs, atrial septum, the tricuspid valve ring, the mitral valve ring, and the fossa ovailis. Colman et al. (2013) have also published a similarly comprehensive model of the whole human atria incorporating both local heterogeneities and AF remodeling. **Figure 7** shows examples of 3D atrial models constructed via regional segmentation and incorporating heterogeneous AP morphologies (**Figure 7A**). Segmentation into different regions is often carried out manually based on known anatomical features.

Given the relative abundance of animal data sources, computational models of animal atria anatomy and electrophysiology are an important tool for studying arrhythmia mechanisms. Therefore, models of other animal species have also been developed, such as the rabbit atrial model from Aslanidi et al. (2009a), the canine models from Colman et al. (2014) and Varela et al. (2016), and the sheep model from Butters et al. (2013). All these models have contributed to further elucidating of the mechanisms underlying atrial arrhythmogenesis, and exemplify the importance of considering models of other animal species, integrating available experimental data, in studies of AF mechanisms and in the discovery of novel therapeutic approaches (Nishida et al., 2010). Other 3D models of human atria developed in recent years have been reviewed in Dössel et al. (2012).

#### Importance of Modeling Heterogeneities in AF Studies

Several modeling studies have shown the importance of considering realistic anatomical structures, fiber orientation, and AP heterogeneity in the initiation and maintenance of reentry in both human (Seemann et al., 2006; Colman et al., 2013; Krueger et al., 2013; Luca et al., 2015; Zhao et al., 2017) and animal models (Aslanidi et al., 2009a; Butters et al., 2013; Varela et al., 2016). Studies have shown the role of anisotropy, mainly due to fiber orientation, in maintenance of AF, and the role of electrical heterogeneity in the initiation of AF (Butters et al., 2013). In particular, it has been shown that the abrupt anisotropy in fiber orientation between the posterior LA and the PVs is critical for wave break leading to reentry (Klos et al., 2008).

Butters et al. (2013) were the first to investigate computationally the mechanisms of initiation and maintenance of AF by describing the individual contributions of electrical heterogeneity and anisotropy, employing an anatomically detailed model of the sheep atria with regional AP variation. This study confirmed that the abrupt changes in tissue anisotropy between the LA and PVs provide an important AF substrate. This was primarily due to the complexity of the fiber structure of the PV region and the RA (in particular, the CT and PMs), as compared to the LA, which is relatively homogeneous. More recently, Zhao et al. (2017) extended human 3D models by incorporating transmural fibrosis, atrial wall thickness, and 3D myofiber architecture, based on *ex vivo* functional and structural imaging of the atria. This study found that the structural characteristics of regions driving AF were characterized by intermediate wall thickness and fibrotic density, as well as twisted myofiber structure.

Although data supports the involvement of atrial fibrosis in the development of AF, whether this is a cause or consequence of AF is still an open question (Schotten et al., 2016). A study on post-mortem human samples from several locations of the atria supported the existence of a correlation between the extent of atrial fibrosis and fatty tissue infiltrations, and the development and severity of AF (Platonov et al., 2011). As described in the previous section, simulation studies have contributed with some insight into the role of fibrosis in AF development. For example, Maleckar et al. (2009a), showed that CM excitability, repolarization, and rate-adaptation properties are strongly modulated by CM-myofibroblast electrotonic coupling, in particular the strength of coupling, number of coupled myofibroblasts, and the pacing rate. These findings suggest that myofibroblast proliferation during structural remodeling may exacerbate repolarization heterogeneities and decrease tissue excitability, thus facilitating abnormal conduction patterns (e.g., conduction block) and the development of a reentrant substrate.

McDowell et al. (2013) included the effect of fibrotic lesions on the initiation and progression of AF in a whole atrial model, finding that atrial fibrosis contributes to dispersion of APD due to gap-junction remodeling, as well as to the proliferation of myofibroblasts. The study showed that the latter was a sufficient condition for unidirectional conduction block following an ectopic beat from the PV region, while myofibroblast proliferation in the fibrotic region was sufficient to trigger reentry. In agreement with the previous study by Maleckar et al. (2009a) they found that the presence of myofibroblasts in the fibrotic region caused alterations of the transmembrane potential, in particular, shortening of APD and elevation of RMP, and these changes were exacerbated by the presence of collagen deposition. However, their proposed mechanism by which myofibroblasts cause inhomogeneous conduction slowing was through the remodeling of the potassium currents responsible for the repolarization phase of the AP, rather than by electrotonic effects resulting from the formation of direct connections between the myofibroblasts and CMs. Another computational study from the same group including the effects of atrial fibrosis, concluded that initiation of AF is independent of pacing location, and instead depends on the distance between the pacing location and the closest fibrotic region (McDowell et al., 2015).

Although much is still unknown about the role of structural and electrophysiological heterogeneities in AF, computational studies have contributed to the systematic characterization of the mechanisms of arrhythmogenesis. In some cases, these have highlighted the importance of patient-specific aspects for clinical therapy, as, for instance, the role of patient-specific fibrosis patterns for guiding catheter ablation procedures (McDowell et al., 2015; Boyle et al., 2016; Deng et al., 2017; Cochet et al., 2018).

## Computational Pharmacology in AF

### Lessons From Existing Rhythm Control Strategies in AF

The available compounds and therapeutic guidelines for AF cardioversion provide important context for AF drug design, and highlight key points of lacking knowledge that may be aided by computational approaches. Effective compounds include class III [amiodarone/dronedarone, intravenous ibutilide, vernakalant (in Europe), dofetilide, and sotalol], class Ic (flecainide and propafenone), and class V agents (cardiac glycosides). Computational studies have addressed their predominant modulatory targets: potassium channels, sodium channels, and NKA, respectively. Computational contributions to increase functional and mechanistic understanding of clinically relevant antiarrhythmic drugs are listed in **Table 1**. Most often these compounds are administered intravenously in the early stages of AF to achieve cardioversion, but flecainide and propafenone are also used orally as pill-in-the-pocket strategies. A critical consideration for choosing among these options is whether structural disease is present. Flecainide and propafenone are contraindicated for all NYHA heart failure classes, while ibutilide and vernakalant are inappropriate for patients with class III-IV disease. These specific characteristics of therapy and contraindications provide an important general hierarchy for understanding the links between the mechanisms of drug action and their clinical utility in AF.

**Table 1:** Summary of AF drugs, ionic targets, and related computational work.

Drug	Class	Target	Computational work
Flecainide	Ic	I_Na_ (Roden and Woosley, 1986) I_to_, I_Kur_, I_Kr_ (Tamargo et al., 2004)	Functional:
			- Flecainide and lidocaine state specific binding models incorporating detailed voltage- and pH-dependence (Moreno et al., 2011)
			Structural:
			Gómez et al. (2014) and Melgari et al. (2015)
Propafenone	Ic	I_Na_, I_to_, I_K1_, I_K_ (Duan et al., 1993) I_to_, I_Kur_, I_Kr_, I_Ks_ (Tamargo et al., 2004) I_K,2P_ (Schmidt et al., 2013)	Functional:
			- State-specificity and kinetics of binding via genetic algorithm search (Pásek and Simurda, 2004)
			Structural:
			Gómez et al. (2014) and Ngo et al. (2016)
Amiodarone	III	I_Kr_, I_Ks_, I_to_, I_Kur_ (Tamargo et al., 2004) I_Na_, I_Ca_ (Nattel et al., 1992) I_K,2P_ (Gierten et al., 2010)	Functional:
			- Multi-target modeling of drug action in AF via Hill-type conductance-only block (Loewe et al., 2014)
			- Pharmacodynamic modeling of drug–drug interactions (Chen et al., 2015)
			- Effect of pharmacologically altered I_Na_ kinetics on post-repolarization refractoriness and APD prolongation (Franz et al., 2014)
			- Mechanistic understanding of amiodarone effects in 1D and 3D, focus on QT prolongation (Wilhelms et al., 2012)
			- Amiodarone targeting I_NaL_ in failing human myocardium simulations (Maltsev et al., 2001)
			Structural:
			Zhang et al. (2016)
Dronedarone	III	I_Kr_, I_Ks_, I_K1_, I_Na_, I_CaL_ (Gautier et al., 2003) I_Kr_, I_Ks_ (Tamargo et al., 2004)	Functional:
			- Frequency and concentration dependent effects in cAF remodeled hearts (Loewe et al., 2014)
			- Drug–drug interaction dronedarone (Denisov et al., 2018)
Ibutilide	III	I_Kr_ (Tamargo et al., 2004)	Functional:
			- Clinical intervention with ibutilide linked with simulated phase synchrony between tissue regions (Vidmar et al., 2015)
Vernakalant	III	I_to_, I_Kr_, I_Kur_, I_K,ACh_, I_Na_ (Fedida, 2007) I_K,2P_ (Seyler et al., 2014)	Functional:
			- Multi-target, cellular mode of action (Loewe et al., 2015)
			- AF termination simulated by I_Na_ block with rapid dissociation through decreased wavebreak and blocked rotor generation (Comtois et al., 2008)
			Structural:
			Eldstrom and Fedida (2009)
Dofetilide	III	I_Kr_ (Tamargo et al., 2004)	Functional:
			- Multiscale cardiac toxicity (TdP risk) predictor (Costabal et al., 2018)
			- Contribution of fibroblasts to cardiac safety pharmacology (Gao et al., 2017)
			- Interaction of hERG channel kinetics and putative inhibition schemes in long QT syndrome (Romero et al., 2014)
			- New hERG Markov model including drug-binding dynamics for early drug safety assessment (Di Veroli et al., 2012)
			- Gender and age on dofetilide induced QT prolongation (Gonzalez et al., 2010)
			Structural:
			Dux-Santoy et al. (2011), Saiz et al. (2011) and Varkevisser et al. (2013)
Sotalol	III	I_Kr_ (Kpaeyeh and Wharton, 2016) I_K_ (Carmeliet, 1985)	Functional:
			- Prediction of drug effects at therapeutic doses in controlled clinical trials and real-life conditions (Chain et al., 2013)
			- Identifying total area of the ECG T-wave as a biomarker for drug toxicity (Jie et al., 2010)
			Structural:
			DeMarco et al. (2018)
Ranolazine	I, anti-anginal drug	I_NaL_, late I_Ca_, peak I_Ca_, I_NCX_, I_Kr_, I_Ks_ (Antzelevitch et al., 2004)	Functional:
			- Antiarrhythmic drug effect specifically in inherited long-QT syndrome and heart failure-induced remodeling (Moreno et al., 2013)
			- Prevention of late phase-3 EADs (Morotti et al., 2016)
			- Combined antiarrhythmic and torsadogenic effect of I_NaL_ and I_Kr_ block on hV-CMs (Trenor et al., 2013)
			Structural:
			Du et al. (2014)
Cardiac glycosides (digitalis compounds)	V	NKA (Vivo et al., 2008; Schmidt et al., 2018) Vagal stimulation (Falk, 1991)	Functional:
			- Effect NKA on cell and tissue refractoriness and rotor dynamics (Sánchez et al., 2012)
			- Physiologically based PK model (Neuhoff et al., 2013)
			- Two compartment PK-PD model for clinical dosage effect (Jelliffe et al., 2014)
			Structural:
Weiss (2007)


First, agents with QT prolonging actions are broadly contraindicated, as even relatively subtle effects on ventricular APD limit their use in AF treatment due to high comorbidity with structural disease. Additionally, Na^+^ channel antagonists may be effective if they do not simultaneously reduce the atrial ERP or increase QT (thus eliminating both classes Ia and Ib). It is probable that the efficacy of class Ic agents is largely due to prolonged ERP accompanying their slow dissociation kinetics, which promotes termination of spiral wave reentry (Comtois et al., 2005; Kneller et al., 2005), but they may also limit triggering ectopy particularly early in disease development (Watanabe et al., 2009; Liu et al., 2011). Lastly, non-cardiac contraindications and drug interactions are a significant consideration, particularly for anticoagulant therapy, a core prophylactic for AF-induced thrombosis.

Given these core characteristics of drugs with established efficacy, major current AF strategies can be classified into two broad groups: (1) those that focus on extending the atrial ERP through atria-specific K^+^ channel targeting, (2) combined therapies that leverage multi-target outcomes and minimize contraindications. In the case of ERP modulators, the key challenge is to improve atrial selectivity and reduce non-cardiac contraindications. For this reason channels that contribute to atrial repolarization but have little role in the ventricle (e.g., I_Kur,_ I_K,Ca,_ I_K,Ach_) are the most attractive targets. Approaches targeting Ca^2+^-handling are largely mechanism-driven, and address disturbances to calcium homeostasis, particularly via RyR hyperactivity and calcium overload secondary to dysregulated Na^+^ homeostasis and CaMKII signaling (Heijman et al., 2015). Finally, recently developed multi-target therapies particularly seek to combine the efficacy of Na^+^ channel blockade with repolarization modulators to generate ideal compound profiles (Ni et al., 2017).

Computational approaches are being applied to all of these avenues. Below, we briefly review major computational methods applied to rational drug design, and then highlight approaches where simulations may be crucial in integrating information taken from high-throughput screening, and traditional *in vitro* and *in vivo* electrophysiology.

### Pharmacological Modeling Approaches

Modeling drug interactions can start at the molecular level with molecular docking or dynamic simulations to test small molecule binding sites and structural protein changes (see **Table 1** for structural modeling contributions for AF drugs). These approaches are attractive because they permit estimation of binding affinities (and kinetics in some cases) based on available 3D-structures. However, the availability of these structures has traditionally been quite limited for ion channel targets, largely due to difficulties in crystallizing transmembrane proteins. With recent advances in cryoelectron microscopy, structure-based drug discovery for integral membrane targets is quickly gaining traction (Lengauer and Rarey, 1996; Shoichet et al., 2002; Meng et al., 2011; Yarov-Yarovoy et al., 2014), and is thought to hold great promise as a support for drug development in the future.

Besides modeling molecular binding sites, one may focus on functionally driven drug–ion channel interactions, based on the classical Hodgkin and Huxley (HH) formalism or more recent Markov modeling formulations. For both HH and Markov formulations, the foundation for modeling the effects of antagonists on observable myocyte electrophysiology has been driven by two major frameworks for conceptualizing drug-binding: (1) the “modulated receptor” hypothesis (Hille, 1977), and (2) the “slow inactivation” hypothesis (Khodorov, 1979). The modulated receptor hypothesis states that drug-binding exhibits selectivity for the functional states of the channel, and that this selectivity can be readily expressed by different association and dissociation kinetics (and resulting affinities) for drug binding to each state (Hondeghem and Katzung, 1977; Hondeghem, 1987). Conversely, the synergistic-inactivation hypothesis is essentially an allosteric mechanism, which suggests that a drug does not need to exhibit selective binding for specific functional states, but instead, once the drug is bound it promotes transition to states in which channels are non-conducting. This mode of block has become somewhat popular for calcium channels (Hering, 2002). Broadly speaking, the modulated receptor hypothesis has been more often applied across various drug–channel interactions, and modified to include the popular “guarded receptor” derivatives. Structure-based modeling will surely refine the application of these approaches in coming years.

Because the states are defined by function, these approaches are largely independent of protein structure, but through the binding kinetics can incorporate both time- and voltage-dependent characteristics of drug interaction. Of course, such approaches require well-defined functional models representing the baseline function of the channel target, as well as any disease-related alterations to channel conductance and gating. Markov models of ion channels have the advantage of being able to more accurately represent inter-dependence of state transitions which can considerably impact the outcome of drug binding simulations. However, more complex Markov formulations are often subject to insufficient data or contradictory parameterization requirements when trying to fit multiple experimental data sets.

Drug modeling with intended clinical application need to take pharmacokinetic (PK), additionally to pharmacodynamic (PD), modeling into account. The focus of this review is on PD modeling, the mechanism and effect of drugs, but the spread of drugs and off-target effects should be acknowledged. For recent reviews on (multiscale) PK/PD modeling in systems pharmacology and drug-induced cardiovascular effects (Collins et al., 2015; Clancy et al., 2016; Danhof, 2016). A good example of the importance is the conversion of drug from amiodarone to its derivative dronedarone. Both drugs share the main structure (removed iodine and added methanesulfonyl group) and electropharmacologic profile, with different relative effects on individual ion channels (Pamukcu and Lip, 2011; Rosa et al., 2014). Amiodarone accumulates in tissue due to a longer half-life and iodine is known to negatively affect thyroid function (Cohen-Lehman et al., 2010). The development of amiodarone to dronedarone was motivated by PK and off-target PD effects, to optimize drug efficacy and limit clinical side effects. PK/PD modeling should be taken in to account for drug development, but should be preceded by establishing the effect of targeting (a combination of) ion channels as possible drug targets.

### Modeling Specific Ion Channels as Drug Targets

As mentioned above, various potassium channels are remodeled during AF and several of them are almost only expressed in the atria (I_Kur_, I_K,ACh_, I_K,2P_, and I_K,Ca_) (Ravens and Christ, 2010; Hancox et al., 2016). Pharmacological inhibition of these channels prolongs the AP and therefore extends the atrial ERP. **Table 2** summarizes which ion currents are included in published the hA-CM models. The majority of clinically relevant drugs, targets or affects I_Na_ and/or I_NaL_ as antiarrhythmic strategy. Below, we focus on computational approaches to the pharmacological modeling of sodium channels and the range of potassium channels that are still considered viable targets for AF rhythm control.

**Table 2:** Summary of ion currents included in the hA-CM models.

Model (reference)	I_K,ACh_	I_bCl_	I_Cl,Ca_	I_f_	I_K,2P_	I_K,Ca_	cAF variant
Courtemanche et al., 1998							
Colman et al., 2013							X
Colman et al., 2016							
Nygren et al., 1998							
Maleckar et al., 2009b	X						
Koivumäki et al., 2011				X			
Koivumäki et al., 2014b				X			X
Skibsbye et al., 2016	X			X		X	X
Grandi et al., 2011	X	X	X				X
Voigt et al., 2013a	X	X	X				X
Schmidt et al., 2015	X	X	X		X		X
Voigt et al., 2013b	X	X	X				X


*Ion currents that are common to all models include: I_*Na*_, I_*CaL*_, I_*to*_, I_*Kur*_, I_*Kr*_, I_*Ks*_, I_*NKA*_, I_*NCX*_, I_*PMCA*_, I_*bNa*_, and I_*bCa*_. Those models that have a comprehensive cAF variant are marked in the right most column.*

#### I_Na_

The dynamics and pharmacologic properties of the cardiac sodium current are among the best-characterized of any electrophysiologic drug target. I_Na_ inhibitors, first quinidine and then the local anesthetics, were observed to have antiarrhythmic efficacy in the first half of the 20th century, and were adopted for treatment well before their molecular actions were known (Nattel, 1993; Rosen and Janse, 2010). Once modern voltage and patch clamp techniques were developed, pharmacologic properties such as state-specificity and association–dissociation kinetics were extensively characterized for a number of compounds (Bean et al., 1983; Rosen and Janse, 2010). The ability of class I compounds (particularly Ic) to suppress premature ventricular complexes spurred the beginning of major clinical trials (CAST 1 and 2, IMPACT) to assess their overall efficacy. The overwhelming failure of these trials (treatment-induced mortality), has driven scientists and clinicians to reconsider both the key pharmacologic characteristics of major antiarrhythmics (particularly I_Na_ antagonists), and the antiarrhythmic classification systems used to guide clinical development (Rosen and Janse, 2010). Computational approaches have been key for understanding several major dynamical characteristics that determine the positive and negative outcomes of I_Na_-targeted drugs in different contexts of arrhythmia.

The modulated receptor and guarded receptor approaches have been essential in understanding the myocyte-level outcomes of I_Na_ antagonists. Early efforts in particular, made useful contributions to distinguish the characteristics of the different subclasses of Na^+^ channel blockers by applying this approach to very simple baseline models of channel gating (Cohen et al., 1981; Bean et al., 1983). The findings of these efforts still define the major characteristics of these subclass distinctions. For example, the role that slow class Ic dissociation kinetics play in determining the utility of this subclass for extending atrial ERP and antagonizing AF is thought to result from the brevity of the atrial AP (Starmer et al., 2003). That brevity in turn prevents fast-dissociating inactive-state antagonists (e.g., class 1b agents) from being effective in AF. This general mechanism of class Ic efficacy is likely to be particularly potent in terminating spiral wave dynamics by expanding both the core and the curvature of the wavefront, thus increasing the size of primary circuits and organizing the fibrillatory pattern (Kneller et al., 2005).

#### I_Kr_

I_Kr_ is expressed in both human atria and ventricles, and its inhibition prolongs APD in both regions. While I_Kr_ block remains a viable strategy for AF targeting, it presents many challenges of ventricular contraindication. Dofetilide is an example of a drug that specifically blocks I_Kr_, and was approved for AF treatment (Elming et al., 2003), but for which safety remains a significant concern (Mounsey and DiMarco, 2000; Abraham et al., 2015; Cho et al., 2017). For this reason, computational approaches are an attractive means for screening compounds with atrial-selective of targeting of I_Kr_, but so far this goal has not been addressed convincingly. Below, we highlight several aspects that should be considered when applying computational approaches to address the role of I_Kr_ antagonists in AF.

The manner in which I_Kr_ targeting compounds promote ventricular AP and QT interval prolongation is a topic of major interest in toxicology screening, and we will not cover it comprehensively here. However, it is worthwhile noting that a classical parameter for characterizing the ventricular arrhythmogenicity of I_Kr_-targeting compounds, reverse-rate-dependence, is also important in atrial drug design. Strong frequency-dependence is highly desirable for AF cardioversion due to very high frequencies of tissue activation during AF. As such, modeling approaches that do not permit accurate assessment of this characteristic are of limited value. To this end, the commonly used Courtemanche model does not reproduce the reverse-frequency-dependency of I_Kr_ block on atrial APD (Tsujimae et al., 2007). By adding a slow activation parameter to the Hodgkin–Huxley model formulation and inhibiting varying combinations of fast and slow gating variables, Tsujimae et al. (2007) reproduced the inhibition dynamics and the frequency dependence of known I_Kr_ blockers (quinidine, vesnarinone, and dofetilide). More recent models have attempted to define I_Kr_ pharmacology in a more detailed manner. For example, Li et al. (2016) first developed a detailed Markov model of I_Kr_ gating, and then embedded it in the O’Hara-Rudy hV-CM model to provide a basis for characterizing compounds with known and varying TdP risk (Li et al., 2017). As a result they found that a mechanism of trapping in the hERG pore (carrier of the I_Kr_ current) represented by an additional Markov state in the pharmacological model, created a better predictability of TdP risk by I_Kr_ inhibitors. Applying models of this detail in atrial and ventricular CM models may provide a basis for better establishing the potential of I_Kr_ blockade for targeting AF. We are not aware that such an approach, especially with the focus on atrial I_Kr_ in AF, has been pursued to date.

#### I_Kur_

Due to atria-specific expression, pharmacological inhibition of I_Kur_ allows for atrial selective APD prolongation with minimal adverse effects in the ventricles (Nattel and Carlsson, 2006). Experimental investigation of I_Kur_ and pharmacological properties is complicated by the lack of drug selectivity and overlap of I_Kur_ block with other currents, such as I_to_ (Ravens and Wettwer, 2011). Furthermore, first clinical trials have controversially shown no decrease in AF burden in patients upon treatment with an I_Kur_ blocker (Shunmugam et al., 2018).

Experimental complications can be overcome by using *in silico* models to assess I_Kur_ involvement in AF and AF treatment. Tsujimae et al. (2008) extended the Courtemanche et al. (1998) I_Kur_ formulation with voltage- and time-dependent pharmacological scaling factor to computationally investigate the voltage- and time-dependent block of I_Kur_ to mimic experimental drug inhibition and effects on AP characteristics. In simulations incorporating AF remodeling, they showed overall APD prolongation for a blocker with fast association kinetics and frequency-dependent APD prolongation when association kinetics were slow, particularly when dissociation was also slow. The same I_Kur_ formulation was used to show that rotor termination in chronic AF depends on binding kinetics of I_Kur_ inhibitors (Scholz et al., 2013).

Computational approaches have also been used to define the kinetic properties of the ideal I_Kur_ antagonist: maximum effect in disease, minimum effect in healthy cells and no (non-cardiac) adverse effects. Ellinwood et al. (2017b) used a six-state Markov model of I_Kur_ fitted with voltage clamp data from hA-CMs and expanded the model with drug-bound states. Incorporating the detailed channel model and drug interactions in the Grandi hA-CM model enabled *in silico* assessment of necessary drug characteristics, showing that drug binding to both open and inactive states yields the largest prolongation of APD and ERP. This inhibition was most efficient at intermediate rates of association, and exhibited similar positive-frequency-dependence independent of binding mode (Ellinwood et al., 2017a,b). These simulations have largely supported the perspective that I_Kur_ is an attractive AF target, and future simulations are likely to be useful for assessing whether the specific binding characteristics and multi-target effects of specific I_Kur_ blockers are capable of realizing this potential.

#### I_K,ACh_

I_K,ACh_ is selectively present in the atria and thus may hold potential as an AF treatment target (Ehrlich et al., 2008). Its response to acetylcholine is decreased in cAF (Dobrev et al., 2001), exhibiting constitutive activity (Ehrlich et al., 2004; Dobrev et al., 2005). Single channel patch clamp experiments of I_K,ACh_ expressed in canine atrial CMs suggest an increase in opening frequency and open probability after tachycardia-induced remodeling, while open-duration, channel conductance, and membrane density were unchanged (Voigt et al., 2007). Bingen et al. (2013) showed that I_K,ACh_ blockade decreased restitution-driven alternans, reduced AF inducibility, and promoted AF termination in rat atrial CM cultures and intact atria. These findings agree with tertiapin block of I_K,ACh_ prolonging ERP and terminating AF in a canine model (Hashimoto et al., 2006).

The importance and involvement of I_K,ACh_ in human atrial electrophysiology and fibrillation is well established, but computational models of this channel are still limited. The models of I_K,ACh_ in human atria are based on various data sources, but show a similar and prototypical involvement in the atrial AP: activation of I_K,ACh_ results in hyperpolarization and pronounced AP abbreviation. Maleckar et al. (2009b) implemented a model of I_K,ACh_ based on patch clamp experiments in canine atria, comprising of current–voltage relationship in combination with a scaling factor depending on half-activation and acetylcholine concentration (Kneller et al., 2002) and extended it with dose dependency. The first model incorporating I_K,ACh_ based on human data was the Grandi model (Grandi et al., 2011), yielding the expected dose-dependent reduction in APD and CaT amplitude with increasing concentration of acetylcholine.

Pharmacological block of I_K,ACh_ in *in vitro* and *ex vivo* experiments showed promising antiarrhythmic effects. However, recent studies have found I_K,ACh_ block to be ineffective both in increasing the left-atrial ERP *in vivo* (Walfridsson et al., 2015) and reducing AF burden in clinical trials (Podd et al., 2016). Pharmacological effects and pathways activated by acetylcholine that are currently not implemented in the existing computational models (e.g., crosstalk with CaMKII and β-adrenergic stimulation) might explain the disagreement between *in vitro*, *in silico*, and clinical studies. *In silico* investigation may help to resolve these discrepancies, and confirm whether this ion channel holds potential as an AF target. Future computational work should address these possibilities, and better describe the effects of regional heterogeneity in I_K,ACh_ expression and acetylcholine release in the atria (Kneller et al., 2002; Jones et al., 2012), as the role of these ion channels in spatial aspects of parasympathetically driven AF remains poorly understood.

#### I_K,2P_

The background potassium current mediated by the TWIK protein-related acid-sensitive K^+^ channel (TASK)-1, a two-pore domain K^+^ channel (K2P), I_K,2P_, has been shown to contribute to APD in hA-CMs (Limberg et al., 2011). TASK-1 encoded by KCNK3 has also been genetically associated with familial AF and early-onset lone AF (Liang et al., 2014). Furthermore, TASK-1 channels are expressed predominantly in the atria (Ellinghaus et al., 2005; Gaborit et al., 2007; Limberg et al., 2011; Schmidt et al., 2015), thus they are a potential atria-specific antiarrhythmic target in AF. However, there is some discrepancy in the direction of association between TASK-1 channels and AF. Some studies reported increased channel expression in cAF (Barth et al., 2005; Schmidt et al., 2015, 2017), while no change was found by others (Ellinghaus et al., 2005; Gaborit et al., 2005). Similarly, functional measurements have shown both increased (Schmidt et al., 2015, 2017) and diminished (Harleton et al., 2015) I_K,2P_ amplitudes in cAF.

The first computational models of I_K,2P_, was published by Limberg et al. (2011). They developed a three-state Markov model of the TASK-1 channel with voltage-dependent transitions between the two closed states, and one open state. The channel model was further integrated to the Courtemanche et al. (1998) hA-CM model to simulate effects of I_K,2P_ on AP, showing that current block led to increased APD (-13%). More recently, Schmidt et al. (2015) published a channel model with less mechanistic detail, using a Hodgkin–Huxley formulation with voltage-dependent activation/deactivation kinetics and steady-state activation. To simulate the effect of I_K,2P_ on AP, the authors integrated their TASK-1 channel model to the Voigt et al. (2013a) hA-CM model.

Pharmacological block of I_K,2P_ with A293 *in vitro* has been shown to increase APD_90_ by +19% (Limberg et al., 2011), +17% (Schmidt et al., 2015) in sinus rhythm hA-CMs, whereas in cAF the reported have been much larger: +58% (Schmidt et al., 2015) and +74% (Schmidt et al., 2017). These findings match well with dynamic patch clamp results, showing +19% and -16% changes in APD_90_ in sinus rhythm with I_K,2P_ subtraction and doubling, respectively (Limberg et al., 2011). The (patho-)physiological significance of the above data has, however, not been yet corroborated *ex vivo* in AP measurements with human atrial trabeculae; isolated hA-CMs are known to have compromised repolarization reserve (Rajamani et al., 2006). Furthermore, TASK-1 channel is also inhibited by some of the commonly used AF drugs, such as amiodarone (Gierten et al., 2010) and vernakalant (Seyler et al., 2014). There is definitely a need for more comprehensive computational studies, investigating the role I_K,2P_ at different stages of AF.

#### I_K,Ca_

All subtypes of SK (small conductance Ca^2+^-activated potassium channel; SK1-3), carrying I_K,Ca_, have been found in the atria, with SK2 and SK3 exhibiting the most atria-specific expression in human cells (Xu et al., 2003; Tuteja et al., 2005; Skibsbye et al., 2014). SK3 encoded by KCNN3 has also been genetically associated with AF (Ellinor et al., 2010; Olesen et al., 2011). The role of SK channels in AF progression appears especially interesting since recent *in vivo* animal studies have showed that their inhibition can reduce the duration of, or even protect against, pacing-induced AF (Diness et al., 2010, 2011, 2017; Skibsbye et al., 2011; Haugaard et al., 2015). However, some studies have also suggested a pro-arrhythmic effect of SK current inhibition (Hsueh et al., 2013). At the cellular level, SK inhibitors NS8593 and ICAGEN induce APD prolongation in hA-CMs (Skibsbye et al., 2014), supporting a role for SK channels in atrial repolarization and encouraging the development of SK-antagonists as an antiarrhythmic strategy. Indeed, the first clinical trial with an SK inhibitor for AF treatment has recently been announced (NTR7012, compound AP30663^1^).

At the pharmacodynamic level, drug-dependent regulation of SK function has been established for several different drugs, but the mechanisms and binding sites are still being examined (Weatherall et al., 2010, 2011; Dilly et al., 2011). In canine atria, inhibition of the SK channels by UCL1684 or apamin prolonged APD (Rosa et al., 1998). Even though the SK channel is a promising target for AF treatment, most drugs targeting SK channels have been shown to have significant affinity for other ion channels (particularly I_Kr_), and as such have often fallen victim to toxicological exclusion.

The most detailed computational modeling effort of SK channels to date has focused on incorporating dynamics from single channel patch clamp experiments in rat SK2 (Hirschberg et al., 1998). This study established two Markov gating binding schemes, each consisting of four closed and two open states, which differentiated two modes of channel gating associated with different mean open probabilities. These models recapitulate observed kinetic components of Ca^2+^-dependent activation and the varied macroscopic open probabilities of single channels, and thus provide a mechanistic basis for interrogating state-dependent drug interaction with SK. However, a comprehensive understanding of SK channel gating is still lacking, particularly as it relates to heteromeric channels, signaling-dependent effects, and to explain the apparent modal gating observed by Hirschberg et al. (1998). Additionally, in the context of the intact atrial CM, subcellular localization and possible colocalization with calcium sources or regulatory proteins remains largely unknown, and is surely important for constructing realistic whole cell models incorporating SK function (Ren et al., 2006; Dolga et al., 2013; Zhang et al., 2018).

There is currently no cardiac-specific computational model that represents both the complex kinetics and pharmacology of SK channels, and their interaction with cardiac Ca^2+^ dynamics. A detailed computational model of the SK channel would enhance our ability to interrogate both the pharmacologic targeting of SK, and the fundamental physiology of SK currents in the atria and in AF. In combination with a hA-CM model with realistic definition of subcellular Ca^2+^ gradients in healthy and AF CMs, the antiarrhythmic effect of SK channels can be probed and drug development optimized.

### Multi-Target Drug Modeling

The strategies described in the previous section focused on specific potassium channels. While these approaches provide simplicity of interpretation, it is well known that virtually all drugs in clinical use have multiple targets in the therapeutic dose range. In some contexts, these effects are thought to be counterproductive, and in others they appear advantageous. AF is a disease that has been particularly well targeted by the so-called ‘dirty drugs,’ namely amiodarone, dronedarone, and most recently in Europe, vernakalant (**Table 1**). Various research fields take advantage of multi-target drug design to discover new treatment options or targets (Ma et al., 2010; Koutsoukas et al., 2011). In AF and other cardiac diseases, existing knowledge has been largely incorporated in computational models, and provides a strong basis for guiding these multi-target therapies.

In general, amiodarone, dronedarone, and vernakalant are thought to be effective in AF for their ability to prolong the atrial ERP through multiple modes of action (Ni et al., 2017), and also to a lesser extent through inhibiting triggered activity via I_Na_ inhibition. Using these drugs as a base, an effort is now being made both computationally and experimentally to define idealized compounds (or personalized multi-therapy approaches), where dual I_Na_ and I_K_ targeting may yield the best therapies. In the case of vernakalant, the primary potassium current target is I_Kur_, while for amiodarone/dronedarone it is I_Kr_ (Heijman et al., 2016). To this end, recent efforts have established a useful line of computational work to describe what idealized versions of these multi-target schemes may be. First, a key requirement was to understand how to limit adverse effects of Na^+^ channel block by optimizing state-dependent block, as shown by (Aguilar-Shardonofsky et al., 2012). Following this, the same group showed improved theoretical AF selectivity by combining Na^+^ current inhibition with I_Kr_ or putative I_Kur_ inhibition (Aguilar et al., 2015). Combined I_Na_/I_Kr_ block improved atrial selectivity over I_Na_ alone, but still exhibited ventricular outcomes (Aguilar et al., 2015). Both that study, and a subsequent investigation only concerning combined I_Na_/I_Kur_ inhibition (Ni et al., 2017), established that selective I_Kur_ blockade could be combined with idealized I_Na_ block to provide more atria-selective antiarrhythmic properties than is achievable via dual I_Na_/I_Kr_ targeting. Adding some complexity to the clinical interpretation of these approaches, Morotti et al. (2016) applied a detailed Markov model to assess the ability of ranolazine to prevent AF re-initiation by blocking I_Na_ reactivation. Like amiodarone, ranolazine is a known antagonist of both I_Na_ and I_Kr_, and while only the I_Na_ interaction was modeled in that study, their results suggest that multiple types of I_Na_ antagonism should be considered for permitting atrial-selectivity.

Additional novel targets, including those described above, as well as calcium handling targets, such as RyR, are likely to offer additional potential through multi-target approaches, particularly once disease-stage specific aspects of AF pathophysiology are better understood. It is already known that the class Ic compounds flecainide and propafenone have RyR blocking activity, which is thought to contribute to their ventricular efficacy (Watanabe et al., 2009, 2011; Galimberti and Knollmann, 2011). *In silico* models will surely be necessary for integrating and further characterizing these multi-target outcomes, and thereby find the most suitable treatment option and guide drug development for various stages of AF.

## Modeling Variability and Uncertainty at the Cell Level

*In silico* drug-screening studies have typically been based on a mechanistic approach where the effect of drug binding is simulated by altering the conductance or the gating kinetics of the target ion channel, as detailed in the previous section. More recently, studies of the mechanistic effects of drug binding on CM electrophysiology have been combined with approaches that allow incorporating natural variability into CM models. This methodology is based on the previously proposed so-called ‘Population of Models’ (PoMs) approach for the study of arrhythmia mechanisms. Simulation studies incorporating the effect of drugs in populations of ventricular myocyte (Passini et al., 2017) and induced pluripotent stem cell-derived CM models (Gong and Sobie, 2018) have shown that incorporating variability into the modeling pipeline allows for a more robust analysis of model predictions of, for instance, the ionic modulators of proarrhythmic mechanisms, the proarrhythmic effects of disease-related remodeling, and drug binding in cardiotoxicity studies of antiarrhythmic drugs.

### Sources of Variability

As discussed in the section “1D and 2D Models of Electrical Conduction in the Atria,” atrial tissue has natural regional heterogeneity both at the cell and tissue (structural) levels. Furthermore, experimental findings have revealed a wide variability in measured APs and ionic current densities that cannot be attributed to regional variations. This intrinsic variability has been demonstrated in numerous reports of experimental data on atrial electrophysiology, both in healthy and pathological conditions, and spanning from the single cell to organ levels. Variability in experimental data has been observed in electrophysiological measurements of both different individuals (inter-subject) and CMs of the same individual (intra-subject). This arises from several sources, in particular, varying expression levels and post-transcriptional changes of ion channels, leading to variable ionic current densities, and of calcium handling proteins in CMs. Additional variability arises from local differences in cellular morphology and shape, and even from circadian rhythms. For a more detailed overview of sources of variability and uncertainty in experimental measurements and models of cardiac electrophysiology (Johnstone et al., 2016; Muszkiewicz et al., 2016).

### Population of Models Approach

Single cell models are typically constructed by fitting the model to average values of experimental measurements, with the aim of deriving a single representative model. The available experimental data have permitted the development of increasingly detailed and refined mathematical hA-CM models. However, the fact that these models are matched to specific data sources, obtained under different experimental settings, often results in families of models that are overfitted to a single source of experimental data. As mentioned above, electrophysiological properties, such as APD, RMP, and repolarization reserve, may vary substantially between the different model lineages, which raises questions about their applicability in a general setting. The PoM approach thus allows to expand the applicability of single cell models by representing a diversity of phenotypes, and may uncover new emergent phenomena that are not observed in the traditionally single “averaged” model. Another application of PoMs is to perform sensitivity analysis on cell models to uncover the ‘global’ effect of model parameters on arrhythmogenic behavior, such as ectopic activity, and reentry. For further reading on PoMs and sensitivity analysis, we refer the reader to a review published in this same issue (Ni et al., 2018).

In order to capture the variability observed in experimental data, the PoM approach has been proposed for the study of cellular electrophysiological mechanisms. This approach, first introduced by Prinz et al. (2003) to model neurons, and later applied to cardiac cell models by Sobie (2009), generally refers to a set of models sharing the same ionic and molecular formulations, but with variable parameters to reflect observed variability in experimental measurements of the biomarkers. Allowing multiple parameters to vary within large ranges can easily lead to unphysiological models, and thus the PoMs typically need to be calibrated. The calibration step can be experimentally driven, by using experimental data to set the boundaries of maximum and minimum values of electrophysiological biomarkers, more typically AP characteristics. Alternatively, defined biomarker distributions can be used to select models from the population, when experimental data is not available. Different approaches to varying parameters and restraining physiological models in the population have been employed (Muszkiewicz et al., 2016), but most commonly these PoMs are built with the aim at reflecting particular conditions or a specific mechanism of interest.

### Extending PoM to Incorporate Drug Effects and Remodeling

One major advantage of using PoMs is that it allows to study the effect of drugs on a wide range of cellular phenotypes and thus provides a better prediction tool of the effect of drugs on the ionic currents. This can be done both in control conditions, and incorporating drug binding effects. The PoM approach has become part of the routine when assessing drug risk with computational models. It has also been adopted by the CiPA initiative as part of the framework for assessing the risk of TdP development in ventricular CMs under antiarrhythmic drug treatment (Colatsky et al., 2016). The combination of PoM approach and drug binding offers a tool for systematically assessing pro-arrhythmic risk of drugs including inter- and intra-subject variability and tissue heterogeneities. Studies have suggested that comprehensive CM models incorporating variability and uncertainty can provide more robust and reliable arrhythmia risk markers and metrics (Pathmanathan et al., 2015; Passini et al., 2017).

Population of model studies incorporating AF remodeling have also shown interesting differences in the ionic determinants of AP characteristics and rate adaptation in normal and AF remodeling conditions (Sánchez et al., 2014; Lee et al., 2016), supporting the role of ion channel remodeling, and RyR kinetics in the development arrhythmogenic alternans (Chang et al., 2014; Lee et al., 2016; Vagos M.R. et al., 2017). Additionally, PoMs offers a strategy for incorporating single myocyte electrophysiological variability into tissue models, representing “population of tissues,” and assessing the ionic determinants of arrhythmic activity. Liberos et al. (2016) used a population of 3D spherical tissues incorporating patient variability to uncover mechanisms of DF and RM during reentry. They DF and RM to be highly dependent on I_Na_, and I_K1_, while RM was inversely correlated to I_CaL_ conductance. In addition to demonstrating the use of PoMs in uncovering the underlying mechanisms of AF perpetuation, this study suggested a dependency of the efficacy of I_CaL_ blockers on I_Na_ and I_K1_, and provided further evidence for the benefits of a combined drug target approach or multi-target agents in the treatment of AF. In another modeling study, Sánchez C. et al., 2017 used a calibrated PoMs of atrial myocytes to build whole atrial models representing six different AP phenotypes with long and short APD, and studied their effects on reentry dynamics. They found that differences in APD resulted in different activation patterns of fibrillatory activity, such as regularity of reentry, conduction block, and interatrial differences of rotor dynamics indices. Interestingly, partial block of I_K1_, I_Na_, and I_NKA_ promoted a slight increase in wave meandering, activation irregularity, and reentry disorganization, which were more pronounced in the phenotypes with shorter APD at early stages of repolarization. This study shed light on the mechanisms of fibrillatory dynamics in the presence of electrophysiological variability and ion channel blockade, suggesting that prolongation of the early phase of repolarization could be a potential antiarrhythmic strategy and corroborating experimental findings on the pro-arrhythmic effect of I_K1_ and I_Na_ block via rotor destabilization. In another study, the authors used PoMs to replicate experimentally measured effects of nNOS-induced shortening of AP by increasing the conductance of I_Kur_, I_to_, I_K1_, and I_CaL_, showing a more pronounced role of I_Kur_ and I_K1_ over the reminder ionic currents in the altered AP phenotype (Reilly et al., 2016).

**FIGURE 8:**
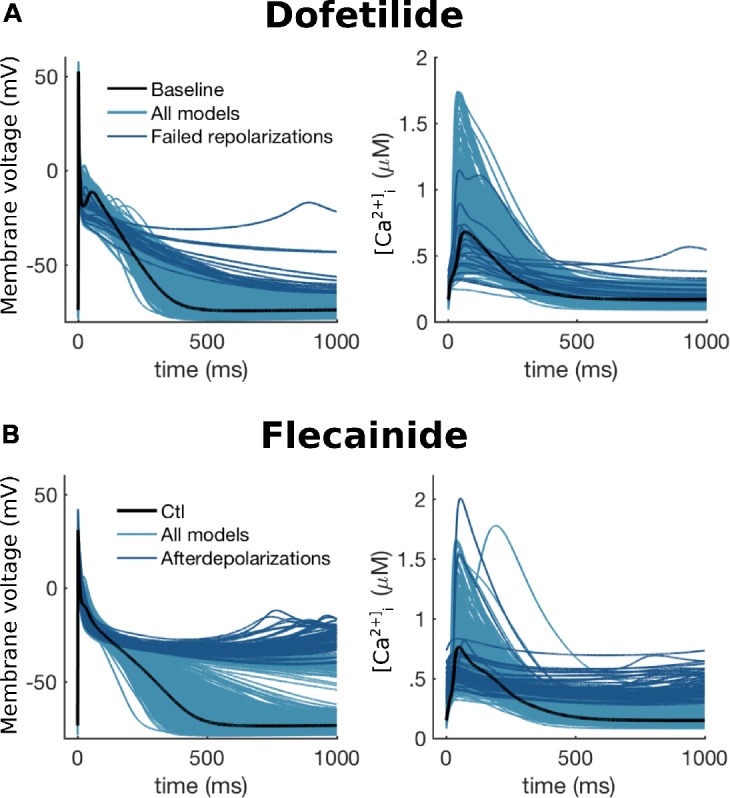
Population of models approach to modeling drug binding effects of dofetilide and flecainide on repolarization abnormalities with an atrial myocyte model. APs (left) and calcium transient (right) traces of dofetilide **(A)** and flecainide **(B)** at 10-fold free plasma concentration are shown. Black lines represent the baseline (control) model, light blue traces represent all models in the populations, and dark blue lines show the models that presented repolarization abnormalities. Simulation results were published in preliminary form in abstract at the Vago M. et al. (2017).

**Figure 8** illustrates the use of the PoM approach in the assessment of the effects of two commonly used drugs in AF rhythm control, dofetilide and flecainide, on repolarization instabilities (here considered as either failed repolarization or afterdepolarizations). The PoM was constructed by varying the density of the major ionic currents in a hA-CM model (Skibsbye et al., 2016) within ±30% following a Gaussian distribution, and the populations were calibrated against experimental data (Sánchez et al., 2014). This study indicated an increased incidence (or probability) of repolarization abnormalities in the populations with both drugs (dark blue to light blue traces ratio), with a 10-fold free plasma concentration, while the baseline (black traces) was mostly unaffected. This example showcases the advantage of using a PoM approach instead of a single averaged model in predicting drug binding outcomes.

Thus, PoMs provide a useful platform for the systematic study of arrhythmia mechanisms at both the single cell and tissue levels, and to obtain a more robust mechanistic insight into, and prediction of drug action on repolarization instabilities, triggered activity, and reentry.

## Concluding Remarks and Future Perspectives

(1) Computational modeling of AF has progressed rapidly in the past two decades and has yielded a body of knowledge surrounding AF disease complexity that could not have been achieved with experimental approaches alone. Although current models are generally oversimplified and computational approaches are not yet truly multiscale with respect to pharmacology, aspects of current approaches, such as idealized drug modeling, are critically involved in the cycle of hypothesis generation and testing.
(2) As described in the section “Computational Pharmacology in AF,” much work still needs to be done in order to develop functionally detailed models of the ion channels thought to offer therapeutic potential in AF. These models, and the cell models in which they are tested, will rely upon new experimental data, not just of the drug–channel interaction but also key aspects of AF pathophysiology, particularly the time course of mechanistic changes during disease progression. New experimental data on the metabolic pathways, such as signaling cascades and phosphorylation of regulatory proteins, involved in remodeling processes and calcium homeostasis dysfunction could be a meaningful addition to hA-CM models. This is especially relevant to models of advanced stages of AF, where pathological phenotype is largely an interplay of several concurrent mechanisms.
(3) Methods that permit a more direct path from experimental characterization to model generation will improve efficiency and constrain uncertainty in the drug–target models. Structure-based approaches may eventually be very useful in this way. Integrated activities of experimentalists and computational scientists will also be essential to determine the most important knowledge for future modeling efforts, particularly as it relates to the stages of AF progression, and personalization. These efforts should be fostered, and cross the boundary between academic and commercial pharmacology.
(4) Personalized approaches will eventually be the ultimate goal of model-based treatment, although in the short-term, applications outside ablation therapy are still relatively distant. Using models as a foundation for developing general rules about the interaction of pharmacologic targeting with geometric characteristics and disease-stage will provide an important intermediate step to the clinic, and one that can be approached in the short to medium term.

## Author Contributions

All authors contributed to drafting the article and reviewing it critically for important intellectual content, as well as, approved the final version of the manuscript.

## Conflict of Interest Statement

The authors declare that the research was conducted in the absence of any commercial or financial relationships that could be construed as a potential conflict of interest.

## Footnotes

1 http://www.trialregister.nl/trialreg/admin/rctview.asp?TC=7012
